# Chemical Composition and Antibacterial Activity of Essential Oils from Different Parts of *Leonurus japonicus* Houtt.

**DOI:** 10.3390/molecules18010963

**Published:** 2013-01-14

**Authors:** Liang Xiong, Cheng Peng, Qin-Mei Zhou, Feng Wan, Xiao-Fang Xie, Li Guo, Xiao-Hong Li, Cheng-Jun He, Ou Dai

**Affiliations:** 1 State Key Laboratory Breeding Base of Systematic Research, Development and Utilization of Chinese Medicine Resources, Sichuan Province and Ministry of Science and Technology, Chengdu 610075, Sichuan, China; E-Mails: xiling0505@126.com (L.X.); zhqmyx@sina.com (Q.-M.Z.); wanfengcdzy@126.com (F.W.); xxf14544@163.com (X.-F.X.); gli64@sina.com (L.G.); lixiaohong136@126.com (X.-H.L.); heye1217@163.com (C.-J.H.); haiou0505@126.com (O.D.); 2 Pharmacy College, Chengdu University of Traditional Chinese Medicine, Chengdu 610075, Sichuan, China

**Keywords:** *Leonurus japonicus*, essential oil composition, antibacterial activity, *β*-caryophyllene

## Abstract

The herb and fruits of *Leonurus japonicus* Houtt., named “Yimucao” and “Chongweizi”, respectively, in Chinese, have been widely used in China as gynecological medicines. The components of the essential oils obtained by hydrodistillation were investigated by GC-MS. The antibacterial activity of the essential oils was determined by micro-dilution assay. The results showed large variations in the chemical composition and antibacterial activity of the oils. The oil of “Yimucao” showed antibacterial activity against various Gram-positive bacteria and consisted mainly of sesquiterpenes and diterpenes, with phytone, phytol, caryophyllene oxide and *β*-caryophyllene being the most significant constituents, whereas the oil of “Chongweizi”, mainly made up of bornyl acetate and aliphatic hydrocarbons, was inactive in the antibacterial assay. Further study of the main compounds in “Yimucao oil” showed that *β*-caryophyllene had wide-spectrum activity against Gram-positive bacteria.

## 1. Introduction

*Leonurus japonicus* Houtt. is an annual herbaceous plant of the Lamiaceae family, widely distributed in East Asia. The herb harvested in summer before blossoming and the ripe fruits picked in autumn, commonly referred to as “Yimucao” and “Chongweizi”, respectively, in Traditional Chinese Medicine, are both used for regulating menstrual disturbances and invigorating blood circulation [1,2]. Previous bioactivity investigations of the *Leonurus* genus, including *L. persicus*, *L. japonicus*, *L. sibiricus* and *L. heterophyllus*, showed that the extracts of aerial parts had anti-inflammatory [3], antibacterial [4,5,6], anti-platelet aggregation [7], and cytotoxic activities [8]. Even more remarkably, two novel proteins from the seeds of *L. japonicus* inhibited the growth of both fungi and bacteria [9,10].

Although “Yimucao” and “Chongweizi” are obtained from the same plant and both are used as gynecological medicines, they are harvested in the culture field, circulated on the market, used in the clinic and recorded in China Pharmacopoeia separately. Generally speaking, “Yimucao” are often used to treat acute nephritis, dispel edema and oliguria, whereas “Chongweizi” can be used as a drug or a tea drink for improving vision and reducing blood pressure [1,2]. Thus, the two medicines from the same plant have something in common and some differences in traditional applications. To illustrate the main differences of the chemical components and the antibacterial activity between “Yimucao oil” and “Chongweizi oil”, and search for the major active ingredients, we used GC-MS to determine the contents of the oils and applied micro-dilution assay to evaluate the antibacterial activity of the oils and the isolates. This paper describes how much difference there is between “Yimucao oil” and “Chongweizi oil” and what the probable antibacterial compositions in the oils are.

## 2. Results

### 2.1. Antibacterial Activity of “Yimucao Oil” and “Chongweizi Oil”

The essential oils were obtained by hydrodistillation from air dried “Yimucao” and “Chongweizi”, respectively, and their antibacterial activity was subsequently assayed by the micro-dilution assay. In our study, 14 standard bacterial strains found in human beings and animals were used, and the MIC values of the oils on these bacterial strains were tested to determine their antibacterial potential (Table 1).

The results showed that “Chongweizi oil” was inactive in the antibacterial assay (MIC > 3.2 mg/mL), while “Yimucao oil” exhibited antibacterial activity against several Gram-positive bacteria, but was ineffective against Gram-negative bacteria. The MICs of “Yimucao oil” were 0.2 mg/mL against *M. caseolyticus*, 0.4 mg/mL against *S. epidermidis* and *E. faecalis*, and 1.6 mg/mL against *S. aureus*, methicillin-resistant *S. aureus*, *S. saprophyticus* and *E. faecium*.

### 2.2. Components of “Yimucao Oil” and “Chongweizi Oil”

The essential oils from air dried “Yimucao” and “Chongweizi” were subsequently analyzed by GC and GC/MS systems. In total, 46 (“Yimucao oil”) and 49 (“Chongweizi oil”) constituents were identified and quantified respectively (Table 2).

**Figure 1:**
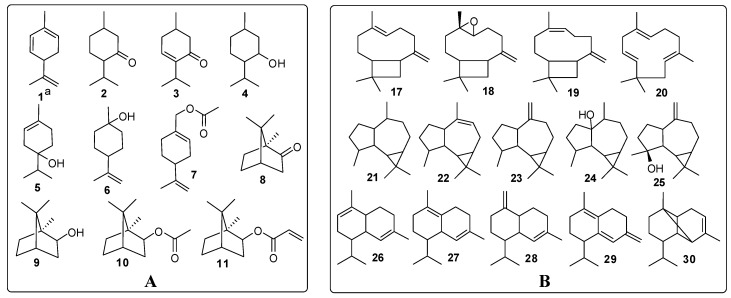
Main monoterpenes (**A**) and sesquiterpenes (**B**) of essential oil from *L. japonicus*.

^a^ The number of the compounds was the same as that in Table 2.

“Yimucao oil” was mainly made up of diterpenes (32.77%) and sesquiterpenes (45.37%), with phytone (19.02%), phytol (13.75%), caryophyllene oxide (11.49%) and *β*-caryophyllene (9.89%) being the most significant constituents. Other sesquiterpenes that were present in appreciable amounts were spathulenol (5.31%), *α*-caryophyllene (3.38%) and isocaryophyllene (3.00%). The main structural types were found to belong to the caryophyllane, aromadendrane and cadinane classes, which accounted for 41.73% of the total oil (Figure 1). In addition, monoterpenes constituted 7.82% of this oil, with a prevalence of menthane and camphane compounds (3.82%), among which oxygenated monoterpenes predominated (Figure 1). Ten aliphatic compounds were found in “Yimucao oil” at low percentages of less than 1%. Unlike the “Yimucao oil”, “Chongweizi oil” was characterized by large amounts of aliphatic compounds (43.04%), including 12 aliphatic hydrocarbons, two aldehydes and five esters. Among them, aliphatic hydrocarbons constituted 32.7% of the “Chongweizi oil”, with the most abundant component being *n*-hexadecane (9.65%), followed by *n*-tridecane (7.72%) and *n*-pentadecane (4.36%), most of which were not detected in the “Yimucao oil”. In addition to aliphatic compounds, the monoterpene fraction was also noteworthy (15.11%), with bornyl acetate (7.33%) and bornyl acrylate (1.81%) as the main constituents, which were absent in the “Yimucao oil”. As regards sesquiterpenes and diterpenes of the “Chongweizi oil”, the fractions were relatively small when compared to the “Yimucao oil”, accounting for only 10.45% and 8.45%, respectively. It is particularly worth mentioning that caryophyllenes, the major compounds of “Yimucao oil”, were present in minor concentrations less than 2% in “Chongweizi oil”. 

Thus, according to the GC and CG/MS analyses, the chemical components of “Yimucao oil” and “Chongweizi oil” were quite different. The “Yimucao oil” was mainly comprised sesquiterpenes and diterpenes, while “Chongweizi oil” was rich in aliphatic compounds, particularly aliphatic hydrocarbons. The result suggested that the significant difference in antibacterial activity of essential oils was relevant to the large variations in the composition.

### 2.3. Antibacterial Activity of the Main Compounds in “Yimucao Oil”

Further investigation on the chemical constituents of “Yimucao Oil” resulted in the isolation and identification of caryophyllene oxide (**18**) [11] and phytol (**38**) [12]. Their structures were elucidated by spectroscopic analyses, including HR-ESIMS and NMR techniques. Subsequent study on the antibacterial activity of the main compounds **17**, **18** and **38** showed their MIC values against nine bacterial strains (Table 3).

Compound **17** showed wide-spectrum activity against Gram-positive bacteria, including *S. aureus*, methicillin-resistant *S. aureus*, *S. epidermidis*, *S. auricularis*, *M. caseolyticus*, *E. faecium* and *E. faecalis*, especially for the last four strains. The MIC values ranged from 0.032 to 0.256 mg/mL, and the MBC values were similar or 2–4 folds higher than MIC values. Compound **18** showed only activity against *S. auricularis*, *M. caseolyticus*, *E. faecium* and *E. faecalis*, while **38** was inactive against Gram-positive bacteria, except for *S. auricularis* and *M. caseolyticus* (MICs, each 0.128 mg/mL).

## 3. Discussion

“Yimucao” and “Chongweizi”, derived from different parts of *L. japonicus*, are two common drugs in Traditional Chinese Medicine. This work provided the first report of the comparison of the chemical composition and bioactivity between “Yimucao” and “Chongweizi”. GC-MS analyses of the volatile constituents showed large variations between “Yimucao oil” and “Chongweizi oil”. The most representative compounds in “Yimucao oil” were sesquiterpenes (45.37%) and diterpenes (32.77%), followed by monoterpenes (7.82%) and a small amount of aliphatic compounds (3.10%). However, “Chongweizi oil” is predominantly composed by aliphatic compounds (43.04%) and monoterpenes (15.11%), followed by sesquiterpenes (10.45%) and diterpenes (8.45%) represent the minor groups in this mixture. The results indicated that the variability in the composition of essential oils depend essentially upon the medicinal parts and the harvest season [13,14].

Then the essential oils also showed a very diverse antibacterial effect. The “Yimucao oil” exhibited antibacterial activity against several Gram-positive bacteria, while the “Chongweizi oil” was inactive in the assay. According to the above results, we could infer that the antibacterial activity of essential oil from *L. japonicus* may be due to the abundant sesquiterpenes and diterpenes rather than aliphatic compounds, especially due to caryophyllenes (accounting for 27.76% of the total oil). Although the “Chongweizi oil” showed no effect against the bacterial strains in our study, innovative researches about “Chongweizi” have led to the purification of two antimicrobial proteins [9,10]. These implied that “Chongweizi” is also an effective antibacterial drug, and the activity may be related to the rich proteins instead of essential oil.

Further investigation on the isolated compounds of “Yimucao oil” showed that *β*-caryophyllene had favorable antibacterial activity against Gram-positive bacteria, with MICs from 0.032 to 0.256 mg/mL and MBCs from 0.064 to 0.256 mg/mL. The results were consistent with the earlier reports [15,16,17]. In particular, *β*-caryophyllene was reported to show significant antibacterial activity against *E. faecium* and *E. faecalis*, the MICs (each 0.025 mg/mL) in the previous investigation were very similar to those in this study (each 0.032 mg/mL). In addition, the antibacterial assay displayed that *β*-caryophyllene was inactive against Gram-negative bacteria, including *Enterobacter cloacae*, *Escherichia coli*, *Moraxella catarrhalis*, *Klebsiella pneumoniae* and *Pseudomonas maltophila*, whereas some reports found *β*-caryophyllene to be active against other Gram-negative bacteria strains, such as *Achromobacter xylosoxidans denitrificans*, *Escherichia coli*, *Chryseobacterium indologenes*, *Citrobacter freundii* and *Flavimonas oryzihabitans*. However, the MICs against *E. coli* were obvious different among the reports (0.625–12.8 mg/mL) [15,16,17,18,19]. This may be the consequence of a problem with the solubility of *β*-caryophyllene, which we also experienced during our study.

## 4. Experimental 

### 4.1. General

GC and GC-MS were measured on an Agilent 7890A/5975C spectrometer. NMR spectra were recorded on a Bruker-AV-400 spectrometer. ESIMS were determined on a Waters Synapt G_2_ HDMS instrument. Column chromatography was performed with silica gel (200–300 mesh, Jiangyou Silical Gel Development Co., Yantai, China), and Sephadex LH-20 (Amersham Pharmacia Biotech AB, Uppsala, Sweden). Mueller-Hinton agar and McFarland Standard were purchased from OXOID (Basingstoke, UK) and bioMerieux Inc. (Marcy-l'Etoile, France), respectively. *β*-Caryophyllene was purchased from J&K Scientific Ltd. (Beijing, China).

### 4.2. Plant Material

The herb of *L. japonicus* (“Yimucao”) was collected in May of 2012 from the field in Wenjiang District, Chengdu City, Sichuan Province, China. The ripe fruits of *L. japonicus* (“Chongweizi”) were gathered in August of 2012 from the same field. Plant identity was verified by Prof. Min Li (Chengdu University of TCM, Sichuan, China). Voucher specimens were deposited at the School of Pharmacy, Chengdu University of TCM.

### 4.3. Volatile Oil Extraction 

Air dried and powdered herb (5 kg) and fruits (5 kg) were separately subjected to hydrodistillation for 10 h using a modified Clevenger type apparatus with a water-cooled oil receiver to obtain the essential oils. The oils were dried over anhydrous sodium sulfate and kept in air tight glass bottles in a refrigerator for further experiments.

### 4.4. Antibacterial Activity Experiments

Three bacterial strains, *S. aureus* (ATCC25923), methicillin-resistant *S. aureus* (ATCC43300) and *E. coli* (ATCC25922) were obtained from the American Type Culture Collection (ATCC, Rockefeller, MD, USA). The other bacterial strains were clinically isolated and obtained from the Teaching Hospital of Chengdu University of TCM. These strains included Gram-positive (G^+^) bacteria: *S. aureus*, *S. epidermidis*, *S. saprophyticus*, *M. caseolyticus*, *E. faecium* and *E. faecalis*; and Gram-negative (G^−^) bacteria: *P. aeruginosa*, *K. pneumoniae*, *M. catarrhalis*, *E. cloacae* and *A. lwoffii* (Table 1). The *in vitro* antibacterial activity was determined by the standard agar dilution method, according to NCCLS (National Committee for Clinical Laboratory Standard) [20]. Volatile oil was dissolved in DMSO to a concentration of 3.2 mg/mL, and then distributed at various concentrations in triplicate with a volume of 100 μL in turbidity tubes. Bacterial suspension (5 μL) with a density of 1 × 10^6^ CFU/mL in Mueller-Hinton (MH) agar was added to each tube. Positive control was a suspension of bacteria in 1 mL of MH agar, and negative control was medium without bacteria. The MIC values (minimum inhibitory concentration at which the microbes failed to grow into a visible spot) were determined after incubation at 37 °C for 24 h.

### 4.5. Analysis of Components

An Agilent 7890A/5975C Gas Chromatography-Mass Spectroscopy system equipped with GC-MSD was used for analysis with ionization achieved by electron impact at 70 eV. The HP-5 MS quartz capillary column (30 m × 0.25 mm, 0.25 μm film thickness) was used with helium (purity 99.999%) as carrier gas at a flow rate of 1.0 mL/min. Experimental conditions for GC analysis of volatile oil were: injection port temperature, 280 °C; column oven temperature, 100 °C for 5 min and programmed at 4 °C/min to 160 °C, kept constant at 160°C for 10 min, then programmed at 5 °C/min to 240 °C; 1 μL injection volume, and split ratio adjusted at 20:1. The mass spectrum of each peak was recorded in the total ion current mode of the mass spectrometer within a mass range of 20 to 800. Identification of oil constituents was achieved using NIST08 mass spectral database.

### 4.6. Isolation of Components from “Yimucao Oil”

The “Yimucao Oil” (4.6 g) was subjected to silica gel CC using a gradient elution of *n*-hexane–EtOAc (100:0–5:1) to afford seven fractions (Fr. A–G). Fr. C was subjected to Sephadex LH-20 (petroleum ether−CHCl_3_−MeOH, 5:5:1) to give four subfractions (C1–C4). The successive separation of C1 with silica gel (*n*-hexane–Et_2_O 50:1) yielded C1-1–C1-6. C1-2 was further purified via PTLC (petroleum ether−EtOAc 15:1) to afford **18** (28 mg), and C1-3 was subjected to PTLC (petroleum ether−EtOAc 8:1) followed by reversed-phase semipreparative HPLC (94% MeOH in H_2_O) to yield **38** (36 mg).

*Caryophyllene oxide* (**18**): Colorless oil; ESI-MS *m/z* 221.2 [M+H]^+^, 243.2 [M+Na]^+^; HRESI-MS: *m/z* 243.1729 [M+Na]^+^ (calcd for C_15_H_24_O, 243.1725); ^1^H-NMR (400 MHz, CDCl_3_) *δ*: 4.96 (1H, brs, H-13a), 4.85 (1H, brs, H-13b), 2.86 (1H, dd, *J* = 10.4, 4.0 Hz, H-5), 2.61 (1H, q, *J* = 7.6 Hz, H-9), 1.19 (3H, s, H_3_-12), 0.99 (3H, s, H_3_-15), 0.97 (3H, s, H_3_-14); ^13^C-NMR (100 MHz, CDCl_3_) *δ*: 51.0 (C-1), 27.3 (C-2), 39.4 (C-3), 59.7 (C-4), 63.8 (C-5), 30.0 (C-6), 29.7 (C-7), 151.8 (C-8), 48.8 (C-9), 39.9 (C-10), 34.1 (C-11), 17.0 (C-12), 112.8 (C-13), 29.7 (C-14), 21.7 (C-15).

*Phytol* (**38**): Colorless oil; ESI-MS *m/z* 319.3 [M+Na]^+^; HRESI-MS *m/z* 319.2978 [M+Na]^+^ (calcd for C_20_H_40_ONa, 319.2977); ^1^H-NMR (500 MHz, CDCl_3_) *δ*: 5.41 (1H, t, *J* = 7.0 Hz, H-2), 4.15 (2H, d, *J* = 7.0 Hz, H_2_-1), 1.99 (2H, m, H-4), 1.67 (3H, s, H_3_-20), 0.87, 0.86 (6H, s, H_3_-18, 19), 0.85, 0.84 (6H, d, *J* = 6.5 Hz, H_3_-16, 17); ^13^C-NMR (125 MHz, CDCl_3_) *δ*: 59.4 (C-1), 123.1 (C-2), 140.3 (C-3), 39.9 (C-4), 25.1 (C-5), 36.7 (C-6), 32.8 (C-7), 37.4 (C-8), 24.5 (C-9), 37.4 (C-10), 32.7 (C-11), 37.3 (C-12), 24.8 (C-13), 39.4 (C-14), 28.0 (C-15), 22.7 (C-16, 17), 19.7 (C-18, 19), 16.2 (C-20).

## 5. Conclusions

It can be concluded that *β*-caryophyllene is in fact the main antibacterial component in “Yimucao oil”. However, it is very difficult to attribute the biological activities of a total essential oil to one or a few active principles, because the minor compounds also may be effective. In addition, it is known that the synergistic or antagonistic effect of a compound present in minor percentage in a mixture has to be considered as well [21]. Thus, further extensive studies on the antibacterial activity of “Yimucao oil” are necessary.

## Figures and Tables

**Table 1:** Antibacterial activity of “Yimucao oil” and “Chongweizi oil”.

NO	Bacterial species	Source	MIC ^a^ (mg/mL)	
			“Yimucao oil”	“Chongweizi oil”
	***Gram-positive bacteria***			
1	*Staphylococcus aureus*	ATCC ^b^ 25923	1.6	— ^d^
2	*Staphylococcus aureus*	CIS ^c^	1.6	—
3	methicillin-resistant *Staphylococcus aureus*	ATCC 43300	1.6	—
4	*Staphylococcus epidermidis*	CIS	0.4	—
5	*Staphylococcus saprophyticus*	CIS	1.6	—
6	*Macrococcus caseolyticus*	CIS	0.2	—
7	*Enterococcus faecium*	CIS	1.6	—
8	*Enterococcus faecalis*	CIS	0.4	—
	***Gram-negative bacteria***			
9	*Escherichia coli*	ATCC 25922	—	—
10	*Pseudomonas aeruginosa*	CIS	—	—
11	*Klebsiella pneumoniae*	CIS	—	—
12	*Moraxella catarrhalis*	CIS	—	—
13	*Enterobacter cloacae*	CIS	—	—
14	*Acinetobacter lwoffii*	CIS	—	—

^a^ MIC: Minimal inhibitory concentration; ^b^ ATCC: American Type Culture Collection (USA); ^c^ CIS: Clinically Isolated Strains, Teaching Hospital of Chengdu University of TCM (China); ^d^ —: MIC > 3.2 mg/mL.

**Table 2:** Chemical composition of “Yimucao oil” and “Chongweizi oil”.

NO	Compounds	RI ^a^	Area (%)	
			Herb oil (“Yimucao”)	Fruits oil (“Chongweizi”)
	***Monoterpenes***			
1	1,5,8-*p*-Menthatriene	1106	0.64	—
2	Menthone	1150	0.34	—
3	*p*-Menth-4-en-3-one	1240	0.57	0.22
4	Menthol	1177	tr ^b^	0.61
5	4-Terpineol	1170	0.41	0.20
6	*β*-Terpineol	1160	0.30	—
7	Perillyl acetate	1438	—	2.17
8	Camphor	1140	0.80	0.43
9	Borneol	1172	0.76	0.66
10	Bornyl acetate	1282	—	7.33
11	Bornyl acrylate	1371	—	1.81
12	Camphene	1066	0.64	1.32
13	*α*-Pinene	936	0.83	0.36
14	*α*-Pyronene	1129	1.59	—
15	*α*-Fenchene	941	0.40	—
16	Elsholtzione	1200	0.54	—
***∑***			***7.82***	***15.11***
	***Sesquiterpenes***			
17	*β*-Caryophyllene	1422	9.89	1.72
18	Caryophyllene oxide	1579	11.49	0.53
19	Isocaryophyllene	1445	3.00	—
20	*α*-Caryophyllene	1452	3.38	0.52
21	Aromadendrane	1465	1.21	tr
22	Dehydroaromadendrane	1460	1.18	tr
23	Aromadendrene	1439	1.31	0.37
24	Palustrol	1588	0.86	—
25	Spathulenol	1573	5.31	—
26	*α*-Muurolene	1495	tr	0.85
27	*δ*-Cadinene	1525	1.73	2.66
28	*γ*-Cadinene	1515	tr	0.67
29	Germacrene D	1480	1.66	—
30	*α*-Copaene	1380	0.71	—
31	*β*-Endesmene	1473	0.68	—
32	*β*-Bourbonene	1383	0.89	—
33	*β*-Patchoulene	1390	—	1.05
34	Patchouli alcohol	1658	0.70	2.08
35	*β*-Cubebene	1350	0.38	—
36	*β*-Elemene	1395	0.21	—
37	Irisone	1460	0.78	tr
***∑***			***45.37***	***10.45***
	***Diterpenes***			
38	Phytol	2100	13.75	2.23
39	Phytone	1830	19.02	tr
40	Isopimara-8,15-diene	1952	—	2.90
41	Dehydroabietane	2078	—	3.32
***∑***			***32.77***	***8.45***
	***Aliphatic compounds***			
42	*n*-Dodecane	1200	tr	1.17
43	*n*-Tridecane	1298	tr	7.72
44	2-Methyldecane	1061	—	0.59
45	*n*-Tetradecane	1401	0.48	1.57
46	*n*-Pentadecane	1500	—	4.36
47	*n*-Hexadecane	1600	tr	9.65
48	*n*-Heptadecane	1706	—	1.01
49	*n*-Octadecane	1799	—	0.37
50	*n*-Nonadecane	1903	—	1.31
51	*n*-Docosane	2200	0.45	2.44
52	Artemisia triene	930	—	2.00
53	1-Dodecene	1195	—	0.51
54	Tetradecenal	1588	tr	0.73
55	(*Z*)-7-Hexadecenal	1806	—	0.78
56	Methyl palmitate	1882	0.93	1.33
57	Methyl isopalmitate	1818	tr	1.33
58	Methyl octadecenoate	2135	0.79	2.61
59	Methyl linoleate	2090	0.45	2.39
60	Methyl linolelaidate	2100	—	1.17
***∑***			***3.10***	***43.04***
	***Other compounds***			
61	1,2,3,4,5,8-Hexahydronaphthalene	1146	—	2.58
62	benzyl benzoate	1732	0.31	0.62
63	Diisobutyl phthalate	1906	—	2.75
64	4-Hydroxy-3-tertbutylanisole	1415	—	0.37
***∑***			***0.31***	***6.32***

^a^ RI: Retention index on a HP-5 MS column; ^b^ tr: trace < 0.1%.

**Table 3:** Antibacterial activity of compounds **17**, **18** and **38**.

NO	Bacterial species	Source	17		18		38	
			MIC (mg/mL)	MBC (mg/mL)	MIC (mg/mL)	MBC (mg/mL)	MIC (mg/mL)	MBC (mg/mL)
1	*Staphylococcus aureus*	ATCC 25923	0.256	0.256	— ^a^	—	—	—
2	*Staphylococcus aureus*	CIS	0.256	0.256	—	—	—	—
3	methicillin-resistant *Staphylococcus aureus*	ATCC 43300	0.256	0.256	—	—	—	—
4	methicillin-resistant *Staphylococcus aureus*	CIS	0.256	0.256	—	—	—	—
5	*Staphylococcus epidermidis*	CIS	0.128	0.256	—	—	—	—
6	*Staphylococcus auricularis*	CIS	0.032	0.128	0.128	0.256	0.128	0.512
7	*Macrococcus caseolyticus*	CIS	0.032	0.128	0.128	0.256	0.128	0.512
8	*Enterococcus faecium*	CIS	0.032	0.064	0.256	0.256	—	—
9	*Enterococcus faecalis*	CIS	0.032	0.064	0.256	>0.512	—	—

^a^ —: MIC > 0.512 mg/mL.
